# Prevalence of obesity and associated health risks in soldiers of the German Armed Forces

**DOI:** 10.1186/s12995-024-00411-y

**Published:** 2024-04-15

**Authors:** Lorenz Scheit, Jan Schröder, Selina Will, Rüdiger Reer, Manuela Andrea Hoffmann

**Affiliations:** 1 Clinic I—Internal Medicine, Bundeswehr Hospital Hamburg, Lesserstr. 180, Hamburg, 22049 Germany; 2 Faculty of Psychology and Human Movement Science, Institute for Human Movement Science, Department of Sports Medicine, University of Hamburg, Turmweg 2, Hamburg, 20148 Germany; 3 Institute for Preventive Medicine of the German Armed Forces, Aktienstr. 87, Andernach, 56626 Germany; 4 University Medical Center of the Johannes Gutenberg-University, Langenbeckstraße 1, Mainz, 55131 Germany

**Keywords:** Bundeswehr, Armed forces, Military readiness, Body mass index, Waist circumference, Overweight, Obesity, Military, Health risk factor

## Abstract

**Background:**

Obesity rates are rising in the armed forces of Western democratic countries, impacting military readiness and health. This highlights the need for preventive health risk assessments and countermeasures.

**Methods:**

Using mandatory health examination data from 2018 to 2022, we analyzed the prevalence of obesity, health risks, and associated specific military risk factors (rank and unit) in 43,214 soldiers of the German Armed Forces. Statistical methods included χ^2^ contingencies and binary logistic regressions.

**Results:**

The prevalence of obesity (BMI ≥ 30) was 18.0%. Male soldiers (OR = 3.776) and those with an officer’s rank (OR = 1.244) had an increased chance for obesity. Serving in a combat unit reduced the chance of being obese (OR = .886). Considering BMI and waist circumference, 2.4% of the total sample faced extremely high cardiovascular and metabolic health risks, while 11.0% and 11.6% had very high or high health risks, respectively.

**Conclusions:**

Our data underscore the importance of targeting obesity-related health risk factors in soldiers to ensure their well-being and deployment readiness.

## Introduction

Armed forces of a sovereign state are essential for maintaining the external security of a democratic state. Operationally fit soldiers are characterized by good physical and mental fitness with high performance [1, 2]. Recent aggressive behaviors by authoritarian states in Europe highlight the renewed importance of operationally fit armed forces [3, 4]. Thus, military readiness or adverse conditions like health risks along with body constitutional characteristics are a matter of debate [5–7].

Based on simple epidemiological body mass index (BMI) data, a response of the German government to a request in 2021 regarding operational capability and severe obesity showed that between 2016 and 2020, only 0.8% of applicants were not admitted to military service due to severe obesity [8]. Also, metrics like waist circumference and the related waist-to-height ratio (WHtR) and accompanying health restrictions have only been part of the German Armed forces’ mandatory health examinations since 2018 [9]. More complex body composition analyses being able to estimate body fat or lean body mass are available only for special units in the German Armed Forces due to the costly and time-consuming character.

Increasing obesity in Western industrialized countries has not been without consequences in the armed forces. An increase in body fat percentage leads to reduced physical performance. The challenge for the armed forces in preventing obesity is to identify the causes and successfully implement countermeasures to improve body weight, and fitness, for optimizing human performance. Few studies on the association of operational readiness with constitution, obesity, physical performance, or fitness are available [10, 11].

The few existing studies describe, for example, that medical officers of the German Armed Forces do not differ in constitutional and fitness characteristics from reference values of the general population, or of the armed forces as a whole [12]. In this regard, it has been reported that there are no relevant differences in constitution, fitness, and activity levels between different medical occupational groups in the Hamburg Armed Forces hospital [13]. Regarding BMI based body constitutional characteristics, there was an increased prevalence of being overweight and obese depending on the duration of service in the German Armed Forces, which, however, was still lower in comparison to other armed forces or the general German population [14]. Considering probable health risks in members of the German Armed Forces, a recent study reported constitutional (obesity) and cardiovascular parameters (blood pressure) had similar prevalence to the German civilian population [9]. In a literature search of international studies there were several surveys of British, French, Czech, Swiss and US Armed forces, included various analytics to explain the observed prevalence of obesity [15–19]. Comparable studies of the German Armed forces have not been done.

Since waist circumference data are available for the German Armed forces, reasonable first step may be investigation of the metabolic and cardiovascular health risks based on the combination of BMI and waist circumference [20]. Additional, but not yet available descriptive prevalence, contingency raw data analyses, and regression model-based analyses could be useful to estimate the impact of obesity and associated risk factors. Currently, epidemiological data on classic health risk factors, e.g. socioeconomic status, family state, and life-style parameters, e.g. nutrition and physical activity levels for the German Armed forces are not available, or registries are not accessible. Analyses incorporating accessible data regarding military rank or unit might be a helpful preliminary approach.

The intent of our study is to reduce the current research gap regarding any relationships between body constitution related health risks and obesity among soldiers of the German Armed forces in a reasonably largescale sample population. Being overweight is not necessarily causally related or associated with health risks due to either increased muscle mass or only slightly increased body fat [19]. Our investigation is focused exclusively on obesity with a BMI greater than 30 kg/m^2^.

The purpose of the present study was (1) to identify the prevalence of increased cardiovascular and metabolic health risks using the combination of obesity related BMI and waist circumference data [20], (2) to determine if there are any associations between obesity and the military rank (officers vs. non-officers) or military unit (combat vs. non-combat), and finally (3) to estimate the impact of the military rank and unit on the obesity status among soldiers of the German Armed Forces.

## Materials and methods

### Data processing

Due to data availability our data was obtained from several databases available at the Institute for Preventive Medicine of the German Armed Forces.This data included almost half a million Bundeswehr staff personal, and was combined in order to achieve individually paired demographic variables (sex, age, height, weight, waist circumference) and army specific data (military rank, military unit) which resulted in a remaining data set of 43.214 individuals (that had been recorded in the period from January 2018 to December 2022). Other variables such as family status, educational level or any diagnosed diseases or syndromes were not available.

BMI was assessed during mandatory routine medical examinations (the so-called “Allgemeine Verwendungsfähigkeitsuntersuchung auf individuelle Grundfertigkeiten” / AVU-IGF which is carried out regularly every three years) [9, 21].

The BMI was calculated from body mass (kg) and body height (cm), which were measured by educated military medical personnel using calibrated scales and tape measures following standard procedures at the nearest 0.1 kg and 0.1 cm, respectively. According to the National Institute of Health & Clinical Excellence (NICE) guidelines [20] in line with the World Health Organization (WHO) recommendations [22], BMI and waist circumference data were classified as normal weight (BMI < 25 kg/m^2^), overweight but not obese (BMI ≥ 25 – 29.9 kg/m^2^) or obese (BMI ≥ 30 kg/m^2^) with obesity being subdivided into obesity class 1 (BMI ≥ 30 – 34.9 kg/m^2^), class 2 (BMI ≥ 35 – 39.9 kg/m^2^), and class 3 (BMI ≥ 40 kg/m^2^). Waist circumference was defined as: Healthy weight (females < 80 cm and males < 94 cm), Overweight (females 80 – 87.9 cm and males 94 – 101.9 cm) and Obesity (females > 88 cm and males > 102 cm). BMI and waist circumference may be associated with obesity related elevated risks, in particular for diabetes type II or coronary heart disease [20].

The military rank for the total of all officers’ ranks, as well as all crew ranks and sergeants’ ranks (non-commissioned officers) were divided into officers and crew (incl. sergeants). The military units were specifically divided into combat units (that may be in direct contact with enemy personnel) and none combat units – including all supporting (replenishment, repair, care) or medical units or any other unspecified units.

Prior to any statistical analyses, incomplete or inconsistent data sets were removed, e.g. if sex was diverse (*n* = 4), missing values for age (*n* = 97), BMI due to missing height or weight (*n* = 1,164), or for missing waist circumference (*n* = 2,933). In the case of repeated measurements for included individuals (e.g. AVU-IGF), the last occasion was considered – meaning that all statistical analyses were conducted as a cross-sectional cohort study.

### Sample

After exclusion of the raw data the remaining sample with complete data sets consisted of 40,165 soldiers (Table 1).

**Table 1:** Descriptive statistics for continuous variables of the total sample (total *n* = 40,165)

		Age (y)	BMI (kg/m^2^)	Waist (cm)
Mean		34.2	26.8	92.1
Standard deviation		8.7	3.7	11.4
Percentiles	25	28.0	24.2	84.0
	50	33.0	26.3	91.0
	75	40.0	29.0	99.0

The total sample may be divided into categories of subsamples for males, females, officers’ rank, non-officer rank (incl. crew and non-commissioned officers), military combat units, non-combat units, and body constitution subsamples (as previously explained) for normal weight, overweight but not obese or obese individuals with obesity subdivided into obesity class 1, 2 and class 3. The weight status was divided into waist circumference clusters, as previously explained, for indicating a healthy weight status, being indicative for overweight, and for obesity (Table 2).

**Table 2:** Frequencies of categorical variables (total *n* = 40,165)

		Counts (n)	(%)
Gender	female	4,167	10.4
	male	35,998	89.6
Military rank	crew	32,808	81.7
	officer	7,357	18.3
Military unit	non-combat	34,719	86.4
	combat	5,446	13.6
BMI (kg/m^2^) weight state	BMI < 25	13,203	32.9
	BMI ≥ 25–29.9	19,718	49.1
	BMI ≥ 30	7,244	18.0
Obesity (kg/m^2^) class 1 - 3	BMI ≥ 30–34.9	6,264	86.5
	BMI 35–39.9	861	11.9
	BMI ≥ 40	119	1.6
Waist (cm) health state adjusted for male/female	normal	21,397	53.3
	overweight	10,122	25.2
	obese	8,646	21.5

Our sample among the Bundeswehr soldiers, consisted of 10.4% females, 18.3% personal with an officer’s rank, and 13.6% soldiers from combat units. Only 32.9% of the soldiers had a normal weight and BMI, but 18.0% had a BMI indicating obesity, and 1.6% for obesity class 3 (BMI ≥ 40 kg/m^2^). For the sex adjusted waist circumference, 21.5% were classified as obese, while 53.3% were classified as normal and healthy.

### Statistical methods

Raw data were presented as mean and standard deviation in the case of continuous variables, or by counts and percentage in the case of categorical variables, respectively. Cross-tabs and the Χ^2^ test were calculated to identify associations between categorical variables. An estimation model was developed to predict the adjusted impact of potentially meaningful variables as related to obesity. The respective binary logistic regression model included sex, age, waist circumference, military rank, and military unit as predictors of obesity. The resulting logistic odds ratio (OR) was interpreted as an increased chance for being obese if the OR > 1, and a reduced chance for an OR < 1, respectively. All statistical analysis procedures were performed with the SPSS version 27.0.

## Results

### BMI and waist circumferences

BMI and waist circumference showed differences in the prevalence of normal, overweight or obese states (Table 2). Cross tabulation analyses for BMI clusters (normal, overweight, obese) and waist circumference clusters (sex-adjusted: normal, overweight, obese) showed body constitutional related health risks. There was an extreme risk for 2.4% of all included cases, and a very high or high risk was found for 11.0% and 11.6%, respectively. For 19.4%, there was an increased risk and 55.6% had no risk with respect to type 2 diabetes and coronary heart disease as related to combined BMI and waist circumference based on the NICE guidelines [13] (Table 3).

**Table 3:**
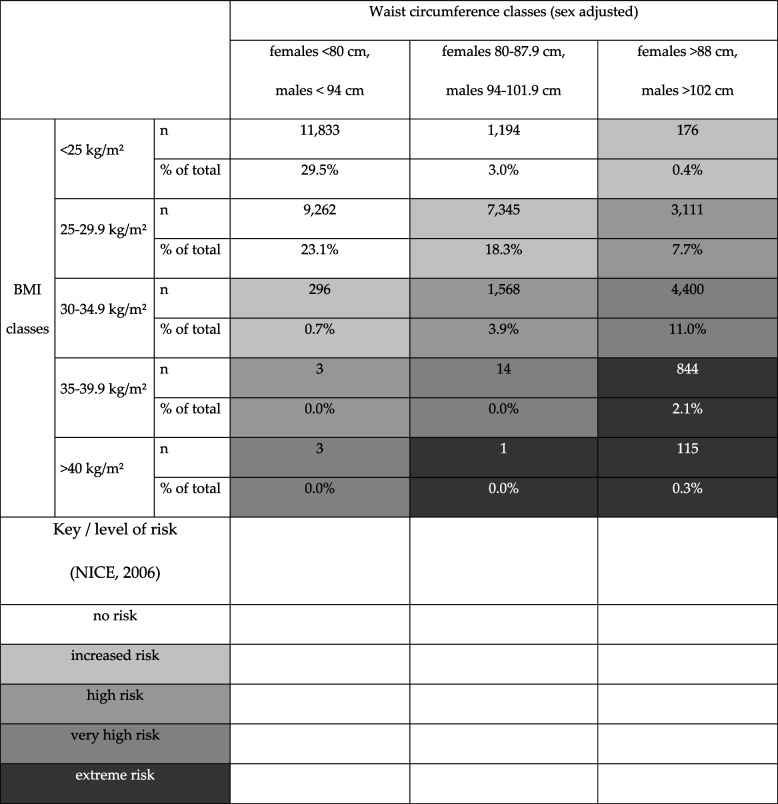
Cross-table of waist circumference and BMI associated health risks (e.g. diabetes or coronary heart disease)

### Obesity

Contingency analyses between obesity (BMI ≥ 30 vs. BMI < 30) and the categorical variables: gender (male vs. female), military rank (officer vs. crew including sergeants’ ranks), as well as military unit (combat vs. non-combat) revealed significant associations (all *p* < 0.001) demonstrated by means of the χ^2^-values (Table 4).

**Table 4:** Contingency table for obesity with gender, military rank and unit (*n* = 40,165)

	Obesity		Total	Χ^2^
	Non-obese	Obese		
Female	3,778	389	4,167	238,184
	90.7%	**9.3%**	100.0%	
Male	29142	6856	35998	
	81.0%	**19.0%**	100.0%	
Non-officer rank	26,643	6165	32,808	68,703
	81.2%	**18.8%**	100.0%	
Officer rank	6,277	1,080	7,357	
	85.3%	**14.7%**	100.0%	
Non-combat unit	28,318	6,401	3,4719	27,503
	81.6%	**18.4%**	100.0%	
Combat unit	4,602	844	5,446	
	84.5%	**15.5%**	100.0%	

These raw data analyses demonstrated that obesity occurred twice as frequently in males (19.0%) compared to females (9.3%). Officers showed a lower prevalence for obesity (14.7%) compared to crew and non-commissioned officers (18.8%). Combat unit soldiers also demonstrated a lower rate of obesity (15.5%) than non-combat soldiers (18.4%).

Analyses for the adjusted impact of continuous and categorical variables on the obesity state are presented in Table 5 (*n* = 40,165). The binary logistic regression model to predict obesity (BMI ≥ 30 kg/m^2^) was significant if gender, age, waist circumference, the military rank, and the military unit were included as predictors (Table 5). The data model explained 59.0% of the total variance (Nagelkerke R^2^ = 0.590), and the correct prediction for obesity was 89.3%.

**Table 5:** Binary logistic regression to explain the chance of being obese (*n* = 40,165)

	B coefficient	SE	*p*-value	Odd’s ratio	Odd’s ratio 95%CI	
					lower bound	upper bound
Gender	1.329	.080	.000	**3.776**	3.225	4.420
Age	-.030	.002	.000	**.970**	.966	.974
Waist	.249	.003	.000	**1.283**	1.276	1.290
Officer’s rank	.218	.053	.000	**1.244**	1.121	1.380
Combat unit	-0.121	.055	.029	**.886**	.795	.988
Intercept	-24.996	.295	.000	.000		

Being male was associated with an increased chance of being obese (OR = 3.776, 95%CI: 3.225–4.420, *p* < 0.001). Being older was associated with a reduced chance of being obese (OR = 0.970, 95%CI: 0.966–0.974, *p* < 0.001). A larger waist circumference was associated with an increased chance of being obese (OR = 1.283, 95%CI: 1.276–1.290, *p* < 0.001). Having a military officer’s rank – in contrast to crew and any non-commissioned officers’ ranks (NCO rank)—was associated with an increased chance of being obese (OR = 1.244, 95%CI: 1.121–1.380, *p* < 0.001). Doing the service in a military combat unit was associated with a reduced chance of being obese (OR = 0.886, 95%CI: 0.795–0.988, *p* = 0.029).

## Discussion

This study evaluated recently available BMI and waist circumference data in order to quantify obesity related metabolic and cardiovascular health risks and to demonstrate categorical associations of obesity and military specific confounders such as military rank and unit. Beside these raw data analyses, the estimated impact of these variables on the obesity status could be explained using binary logistic regression models. In contrast to earlier studies which focused on being overweight and obese and related to the duration of military service [14], our prevalence analyses focused on the obesity rate in the German Armed Forces.

Our results can be summarized as a total obesity prevalence of 18% based on the current BMI criteria, which was markedly higher than reported earlier for the German Armed forces [14]. Referring to either BMI and waist circumference criteria [20], we found extreme, very high or at least highly increased cardiovascular or metabolic health risks of 2.4%, 11.0% and 11.6%, respectively. Only 55.6% of the soldiers demonstrated no risk. The regression model for the adjusted impact of military specific confounders revealed that being part of a combat unit is associated with a reduced chance of obesity (OR = 0.886), while having an officer’s military rank showed an increased chance of obesity (OR = 1.244), which might be misunderstood as contradiction to the officers’ lower obesity prevalence of 14.7% compared to non-commissioned officers and crew ranks (18.8%). This may be better understood if it is taken into account that the odd’s ratio represents an estimate in a logistic regression model with adjustments for several accompanying variables, e.g. sex or combat unit. Thus, the increased chance of obesity in officers is influenced also by the confounding sex with males being at a higher chance for obesity and by the military unit with the majority of soldiers—and of course also officers—belonging to non-combat units, which in turn demonstrate also an increased chance to be obese.

Referring to the above mentioned prevalence of obesity, earlier studies reported a prevalence of 10% (2016–2017) in the French Armed forces, 12% (2014) in the UK Armed forces, and 15% (1999–2009) in the Czech Armed forces [15, 17, 18, 23]. More current data for the U.S. Armed Forces published by Hollerbach et al. in 2022 reported an obesity prevalence of 18.5% [23], similar to our findings of 18%, and which possibly reflects more accurately the current state of increasing obesity prevalence in society in general.

Our findings of BMI and waist circumference associated metabolic and cardiovascular health risks, in 2.4% soldiers being at extreme risk of suffering from metabolic and cardiovascular diseases has provided additional information to other recently reported data on BMI, blood-pressure and physical fitness among soldiers of the German Armed forces [9] These findings emphasize that only 55.6% of all soldiers do not have body constitution-based health risk and highlight the necessity to identify and evaluate appropriate countermeasures in high risk individuals.

With regard to the categorical associations between the obesity status and the military rank or military unit, we found a higher percentage of obese soldiers among crew or non-commissioned officers (18.8%) compared to officers (14.7%), and a lower percentage for obesity within combat units (15.5%) compared to non-combat units (18.4%). Again it should be emphasized that raw data contingency analyses must be distinguished from estimated impacts of variables being adjusted for the presence of other confounders incorporated into regression models. While it might be a little surprising that the regression analysis showed an increased chance of obesity in officers (OR = 1.224), while crosstabulation analyses demonstrated lower incidences for officers, this contradiction between logistic regression (estimates) and contingency analyses (incidences) was not relevant for our findings concerning military unit membership. The reduced chance of obesity in combat units (OR = 0.886) is consistent with the lower prevalence rate mentioned above.

Our findings are also consistent with earlier regression analytic findings for the British and the French Army reported by Sanderson et al. and Quertier et al. [15–17, 23]. In contrast to their findings, age played a minor role in our results.

From a methodological perspective, our study assumes that obesity may be detected appropriately from simple anthropometric parameters, e.g. body mass and height and waist circumference. For epidemiological purposes, the BMI and waist circumference, are assumed to be valid surrogate measures of body constitution associated with cardiovascular and metabolic health risks [20]. Gallagher et al. established a direct link of BMI values to estimated body fat percentages assessed by means of bio-impedance-analyses (BIA) in a multicentric evaluation of large populations covering different ethnicities and found normal body fat percentages for healthy people [24]. Although there are some weaknesses, especially for certain populations. A high BMI might be at least in part, related to increased muscle mass, which has been demonstrated in a very athletic sample of special forces soldiers of the US Army [25]. Our own – so far unpublished – echo-MRI data show body fat percentages ranging from 2.4% to 20% among special forces soldiers, who would have been classified as overweight according to BMI criteria (BMI ≥ 25 but < 30 kg/m^2^). However BMI might not be a sufficiently sensitive measure for body fat monitoring after athletic training interventions [26, 27]. Additionally, being overweight as indicated by increased BMI may be characterized, at least in part by slightly increased body fat, which may not be linked to an increased cardiovascular or metabolic health risk. A slight increase BMI (≥ 27.5–29.9 kg/m^2^) might even be health protective against certain comorbidities among older adults [26–28]. Being underweight or overweight are associated with increased mortality. This relationship is reflected in a J-curve whose optimum lies at a BMI of 18.5–24.9 [29]. Therefore, we included exclusively obese individuals (BMI ≥ 30 kg/m^2^) for potential health risk estimations [20].

While excess body fat causes concern in the general population in relation to cardiovascular and metabolic illnesses, it poses an additional problem for the military, particularly for military readiness [26, 27]. Furthermore, Simchoni et al. described a negative association between obesity and reduced cognitive performance. Decreased cognitive potential combined with limited physical capability limits military personnel readiness, which negatively impacts the operational capability of the armed forces [3, 4, 30]. The negative impact of obesity on the readiness of soldiers has been reported in several studies, not only in terms of reduced recruitment of suitable individuals, but also in terms of reduced endurance and performance [31].

Countermeasures for obesity should not only involve physical activity and fitness promoting health behavior (e.g. diet, sleep and lifestyle) – among more physically fit soldiers but also to civilian populations with equal BMI [15, 17, 18, 23, 32–34].

If preventive measures for obesity, lifestyle and physical fitness are not implemented there would be a large economic burden related to the cost of treatment for the resulting medical conditions [27]. Gazdzinska et al. studied the health behavior of Polish soldiers and found that normal-weight soldiers with higher education living in big cities showed significantly stronger health behavior than soldiers with lower education living in rural areas who were diagnosed with obesity [35]. The authors recommend increased education on health-promoting behaviors and organized training with psycho-dietary counselling for obese soldiers [35].

Obesity-related health risks affect military readiness. Bornstein et al. studied cardiorespiratory fitness, BMI and injury rates in US Army recruits to assess the impact on military readiness and rated the “hardest-hit” US states, which usually had higher rates of obesity and disease leading to a lower military operational capability [36].

From our point of view, health promotion and forced health protection/prevention [32] is of crucial importance for the prevention of obesity, obesity-related illnesses and injuries and in the health risk assessment of the armed Forces. Health promotion involves raising awareness of the effects of health behavior on soldier and the consequences for the military mission. Additionally, future research should investigate intervention models (reward or advice approaches) where appropriate.

## Limitations

Height, weight and waist circumference data were collected during obligatory military medical examinations at different time points (AVU-IGF) and by different personal, which may result in some inaccuracies in spite of existing standard operating procedures. But the mandatory character of data acquisition prevented a selection bias that might occur in comparable civilian studies due to voluntary participation.

Statistical analyses depended on matched individual data for anthropometric parameters and probably confounding variables from several registries, which led to a large loss of cases possibly increasing the risk of selection bias although the remaining sample of more than 40,000 soldiers was still reasonably large and adequate for statistical analysis.

Unfortunately, we were limited to military specific possibly confounding parameters, such as rank and unit. We had no access to well established health risk factors associated with the socioeconomic status, educational level or life-style components such as smoking, nutritional behavior or physical activity. Thus, we were not able to confirm either the increased impact of obesity by the marital status as reported earlier for the British Army by Sanderson et al. [8–11], or the influence of the socioeconomic status and the level of education in the French Army, or the protective role of physical activity and non-smoking reported earlier by Quertier et al. [17] and Yang et al., who investigated Austrian young conscripts [37].

A significant limitation of this investigation was the inability to obtain data related to socioeconomics, family history, physical activity, health or disease issues, educational level, and lifestyle (e.g. nutrition, sleep or smoking).

## Conclusions

This study provides new information on obesity-related health risks in Bundeswehr soldiers by combining BMI and waist circumference data as a function of occupational factors (rank and unit). The study partially closes a research gap for the specific occupational group of soldiers, as it shows the influence of some external service-related factors on the probability of obesity. Access to additional Bundeswehr databases currently protected by law may be able to further close this research gap and allow improved preventive measures to avoid obesity and to promote the health and maintain the operational capability of soldiers.

The application of preventive measures to avoid obesity and to promote the health and maintain the operational capability of soldiers. Future prospective studies should record further classic health risk factors, which should be included in the Bundeswehr’s mandatory health check-ups.

## Data Availability

The data of the soldiers—for this retrospective observation—were already available within the framework of the legal mandate of the Institute for Preventive Medicine of the German Armed Forces. The data protection regulations result from Article 9 para. 2 j DSGVO in conjunction with § 27 BDSG and § 29a para. 5 no. 1 BDSG. § Section 27 of the Federal Data Protection Act (BDSG) and Section 29a para. 5 no. 1 of the Soldiers’ Act (processing of special categories of personal data). Thus, the use and processing of this data in the context of the preparation of this publication follows the legal provisions of the EU, the Federal Republic of Germany and the special provisions of the German Armed Forces to which all soldiers are subject. Section 29a, paragraph 5, no. 1 of the Soldiers’ Act provides for permission to process health data, biometric data, for purposes of scientific research or for statistical purposes.
